# Bio-Functional Constituents from the Stems of *Liriodendron tulipifera*

**DOI:** 10.3390/molecules17044357

**Published:** 2012-04-10

**Authors:** Chien-Chih Chiu, Han-Lin Chou, Pei-Fang Wu, Hsin-Liang Chen, Hui-Min Wang, Chung-Yi Chen

**Affiliations:** 1 Department of Biotechnology, College of Life Science, Kaohsiung Medical University, 100, Shih-Chuan 1st Road, San-Ming District, Kaohsiung 80708, Taiwan; 2 Department of Fragrance and Cosmetic Science, Kaohsiung Medical University, 100, Shih-Chuan 1st Road, San-Ming District, Kaohsiung 80708, Taiwan; 3 School of Medical and Health Sciences, Fooyin University, 151, Ching-Hsueh Road, Ta-Liao District, Kaohsiung 83102, Taiwan

**Keywords:** *L. tulipifera*, liriodenine, (-)-anonaine, (-)-glaucine, (-)-norglaucine, antioxidant, tyrosinase, melanoma, anti-migration, ROS

## Abstract

Four known compounds have been isolated from the stems of *Liriodendron tulipifera*, and the structures of these pure constituents were determined using spectroscopic analysis. Isolated compounds were screened for free radical scavenging ability, metal chelating power assay and ferric reducing antioxidant power assay (FRAP). The anti-tyrosinase effects of *L. tulipifera* compounds were calculated the inhibition of hydroxylation of L-tyrosine to L-dopa according to an *in vitro *mushroom tyrosinase assay. The study also examined the bio-effects of the four compounds on the human melanoma A375.S2, and showed that liriodenine (**1**) and (-)-norglaucine (**4**) significantly inhibited the proliferation of melanoma cells in the cell viability assay. Wound healing results indicated that liriodenine (**1**), (-)-glaucine (**3**) and (-)-norglaucine (**4**) exerted anti-migration potential. Interestingly, (-)-glaucine (**3**), neither liriodenine (**1**) nor (-)-norglaucine (**4**) showed promising anti-migration potential without inducing significant cytotoxicity. Furthermore, a dramatically increased level of intracellular reactive oxygen species (ROS) was detected from (-)-glaucine (**3**). The cell cycle assessment demonstrated a moderate G2/M accumulation by (-)-glaucine (**3**). The above results revealed the anti-cancer effects of *L. tulipifera* compounds, especially on the anti-migration ability indicating the promising chemopreventive agents to human skin melanoma cells.

## 1. Introduction

The essential influence of diet for the avoidance of human sicknesses is well known [1,2,3]. The natural anti-oxidative properties of plants have curative roles in human physical conditions [4]. The anti-oxidative behaviors of fresh vegetables, fruits and plant extracts have been confirmed to have various useful bio-functions for the prevention of cancer [5], retarding aging [6] or cardiovascular illness [7]. Natural anti-oxidative ingredients are extremely noteworthy in the cosmetics business, agriculture industries or food applications due to their abilities to diminish free radical-mediated degradation of cells and tissues [8,9]. The typical established mechanism involves scavenging of free radicals to reduce the oxidative stress and mitigate the development of human illnesses [10,11]. Fruits, legumes [12], vegetables [13], whole-grain cereals [14] and some other foods constitute importaant sources of natural anti-oxidative components [15]. 

Reactive oxygen species (ROS) are generally defined as reactive oxygen-containing chemical species with free radicals, such as hydrogen peroxide, hydroxyl radicals or superoxide [16]. ROS can be generated through a variety of metabolic pathways in different organisms ranging from bacterial to mammalian cells [17]. Additionally, cancer cells are known to be metabolically active and undergo higher levels of oxidative stress, which are associated with deregulation of cell proliferation and aberrant regulation of signaling [17,18]. Many anti-cancer compounds are reported to contribute to the persistent generation of ROS and consequently cause anti-growth [19], cell cycle arrest [20] or apoptosis effects [21] in cancer cells. Accordingly, anti-cancer therapies are based on the rationale design which triggers the apoptosis of cancer cells by inducing a high level of intracellular ROS [22]. Recently, some researchers have reported that certain compounds exert anti-migration effects in cancer by enhancing the cellular ROS level without inducing significant cytotoxicity. For example, Adhikary’s work showed that the natural compound theaflavin retards the migration of human breast carcinoma cells by inhibiting NF-κB via intracellular ROS-mediated signaling mediated, indicating the role of ROS in anti-cancer treatment [23]. Many anti-cancer therapies are based on killing cancer cells by generating high ROS [22]. It has been reported that the modulation of the redox status in cells could led to apoptosis in breast carcinoma, MDA-MB-231 [24].

Hyper-pigmentations, such as freckles, nevus, senile lentigo, birthmarks, pigmented acne scars and melasma are of serious worry to women and even men [25]. The therapy procedures usually involve medications, medicinal cosmetics possessing skin-whitening ingredients or de-pigmenting agents. Tyrosinases are well-known to be the first two rate-limiting enzymes in the synthesis of the human pigment melanin responsible for coloring skin, eyes and hair [26]. Tyrosinases have unique bio-functions to catalyze two distinct reactions in the pathway of melanin synthesis: the hydroxylation of L-tyrosine to L-dopa and the oxidation of L-dopa to dopaquinone, and after further series of conversions eumelanin or pheomelanin are produced [27]. Consequently, natural tyrosinase inhibitors have medicinal uses to treat dermatological hyper-pigmentation diseases involving the overproduction of eumelanin and pheomelanin, and are significant in cosmetics for skin whitening applications [28]. 

During the past several decades, the incidence of malignant melanoma has been increasing faster than any other types of cancer and it is now the one of most common form of cancer diagnosed in the United States [29,30]. In spite of the fact that overall survival following diagnosis has improved, about one-tenth of melanoma patients still have a low 5-year-survival rate [31]. In clinic, melanoma has such a poor prognosis that recent treatments do not have a great effect on decreasing mortalities and prolonging survivals of melanoma patients [32]. Under physiological conditions, cell migration plays an important role for maintaining development and homeostasis in normal physiological functions [20]. In cancer cells, the deregulation of cellular migration is closely associated with metastasis. Additionally, the hyper-proliferation and metastasis of cancer are the main causes of patient death [20]. There are few reports on the therapeutic treatment of metastatic cancer due to the lack of effective systemic medical agents. The intracellular drug inactivation, resistance to drug-induced apoptosis, decreased drug uptake into the cells, repair of drug-induced damage or increased drug efflux are various mechanisms for human melanoma resistance to treatment [33,34]. Fortunately, several compounds derived from natural products have shown the possibility of improved therapeutic effects against melanoma [20,35,36,37]. 

**Figure 1:**
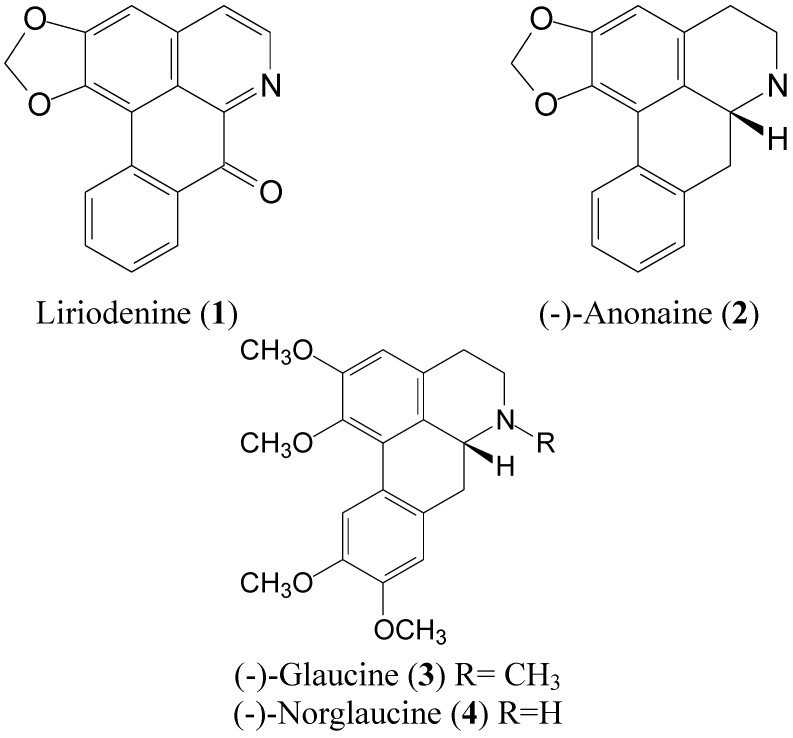
The chemical structures of compounds **1**−**4** from the stems of *L. tulipifera*.

*Liriodendron tulipifera* is one of the fast growing hardwood tree species native to the United States [38]. Previous studies showed that alkaloids were abundant in *L. tulipifera*, which exerted antioxidant activity [38,39]. However, little is known about other bio-functions of this plant. In the study, we investigated the bioactivities of four alkaloid derivatives (Figure 1) isolated from *L. tulipifera*, including their antioxidant activity, tyrosinase inhibition, cellular proliferation, migration and the modulation of intracellular ROS.

## 2. Results and Discussion

### 2.1. Antioxidant Activities and Mushroom Tyrosinase Inhibition of Compounds ***1*** to ***4*** from *L. tulipifera*

The scavenging of 1,1-diphenyl-2-picrylhydrazyl (DPPH) radicals was used in this investigation, as antioxidants act to inhibit this oxidation. In the DPPH study, antioxidants are able to reduce the stable DPPH radicals to the yellow colored diphenyl-picrylhydrazine. Compound **4** presented a minor inhibitory effect of 14.6% at 100 μM in the DPPH assay compared to vitamin C (Table 1). The ferrous ion chelating activities of *L. tulipifera* compounds are also shown in Table 1. Ferrozine quantitatively produced complexes with Fe^2+^. In the presence of chelating reagents, such as the test samples, the complex is disturbed resulting in a lightening of the typical red color. Compound **4** showed a minor level of Fe^2+^ scavenging effect of 14.7% whereas EDTA presented a strong scavenging ability. In the reducing power assay, the color of the test solutions changed from yellow to different shades ranging between green and blue depending upon the anti-oxidative capacities of these antioxidants. The presence of compounds **1** to **4**, similar to the reference antioxidant substances, induced the reduction of the Fe^3+^/ferricyanide complex to the ferrous form. Table 1 summarizes the reducing power of the four pure constituents at 100 μM, which was moderate compared to 3-*tert*-butyl-4-hydroxyanisole (BHA) at the same dose (OD_700_ = 0.98). Next, we measured the inhibitory effect of four constituents in an *in vitro *mushroom tyrosinase inhibition assay (Table 1). Compared to the inhibition of 69.2% displayed by kojic acid that is a commonly used human tyrosinase inhibitor in the cosmetic industry compound **1** showed a medium to minor (20.5%) inhibition of mushroom tyrosinase . 

**Table 1:** Antioxidant activity and the inhibition of *L. tulipifera* to mushroom tyrosinase at 100 μM. (-), no testing; (ns), no significance.

Compounds	DPPH (%)	Chelating (%)	Reducing power (OD700)	Mushroom tyrosinase inhibition (%)
Vitamin C^a^	83.6 ± 1.8	－	－	－
EDTA^b^	－	92.8 ± 4.5	－	－
BHA^c^	－	－	0.98 ± 0.1	－
Kojic acid^d^	－	－	－	69.2 ± 0.1
Liriodenine (**1**)	ns	ns	0.10 ± 0.0	20.5 ± 0.4
(-)-Anonaine (**2**)	ns	ns	0.16 ± 0.0	ns
(-)-Glaucine (**3**)	ns	ns	0.24 ± 0.0	ns
(-)-Norglaucine (**4**)	14.6 ± 3.7	14.7 ± 0.8	0.22 ± 0.0	ns

Data were expressed as a mean value of at least three independent experiments; ^a^ Vitamin C was used as a positive control on DPPH assay at 100 μM; ^b^ EDTA was used as a positive control on metal chelating ability at 100 μM; ^c^ BHA was used as a positive control on reducing power at 100 μM; ^d^ Kojic acid was used as a positive control of mushroom tyrosinase assay at 100 μM; * *p* < 0.05 for standard *vs.* compound.

### 2.2. Anti-Proliferative Properties of Compounds ***1*** to ***4*** from *L. tulipifera* on A375.S2 Cells

**Figure 2:**
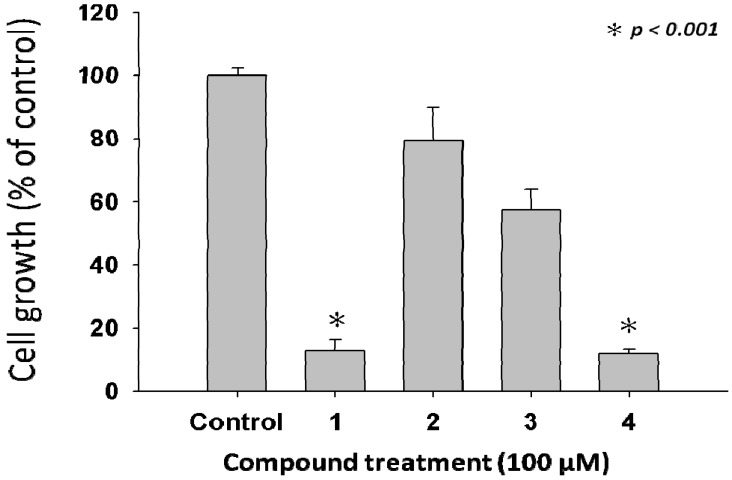
Anti-proliferative effects of *L. tulipifera* compounds on A375.S2 cells. Cell growth was determined by MTT assay after incubation with 100 μM of compounds **1**–**4** respectively. Results are expressed as the percent of the cell proliferation of the vehicle control at 24 h.

Nowadays the main reasons behind the ineffective medical therapies responsible for the high death ratios to cancer patients are from malignant cancer proliferation and metastasis. Therefore, it is vital and valuable for biologists to develop novel medicinal agents for anti-cancer treatment improving the effectiveness of treatment. The melanoma cell line A375.S2 used in the study, is metastatic and widely used in many anti-melanoma studies [20,33]. Accordingly, A375.S2 is suitable as a representative target in the study. The 3-(4,5-dimethylthiazol-2-yl)-2,5-diphenyltetrazolium bromide (MTT) assay was used to investigate the stimulating of cell death caused by the tested compounds. The cell proliferation assay examined the anti-cell proliferation of *L. tulipifera *compounds on human melanoma A375.S2 cells after a 24 h treatment. The samples were treated with 100 μM of four compounds as demonstrated in Figure 2. We observed that cells treated with the tested concentrations of compounds **1** and **4** exhibited more than 50% of cell inhibitory effects for up to 24 h.

### 2.3. Compound ***3*** Causes a Moderate Accumulation of G2/M Phase Population

**Figure 3:**
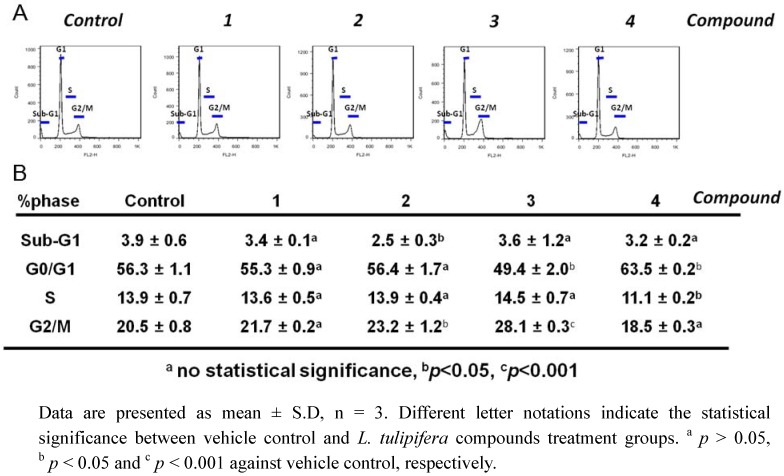
Effects of *L. tulipifera* compounds on cell cycle progressions of melanoma A375.S2 cells. A375.S2 cells were treated with the 100 μM of four compounds for 24 h, respectively. (**A**) Among these four compounds-treated cells, an accumulation of G_2_/M population in compound **3**-treated A375.S2 cells was observed. (**B**) The quantification analysis.

We next examined the effects of the four compounds on cell cycle progression in A375.S2 cells after 24 h of treatment by PI staining followed with flow cytometry. The accumulation of G2/M population is considered as a biomarker for DNA damage and growth inhibition [21]. As presented in Figure 3, compound **3** induced a moderate accumulation of G2/M populations compared with the vehicle control, whereas no significant changes were seen in other compounds’ results. There are studies to show that genotoxic agents cause the G2/M-arrest and eventually cellular apoptosis. For example, costunolide induces G2/M-arrest and apoptosis in breast cancer cells, MDA-MB-231 [21]. However, no significant sub-G1 population was found in compound **1**–**4** treated cells, indicating that the dose at 100 μM of compounds inhibited the proliferation and cellular migration of A375.S2 cells effectively without inducing cell death.

### 2.4. *L. tulipifera* Compounds ***1**–**4*** Attenuated Migration of A375.S2 Melanoma Cancer Cells

**Figure 4:**
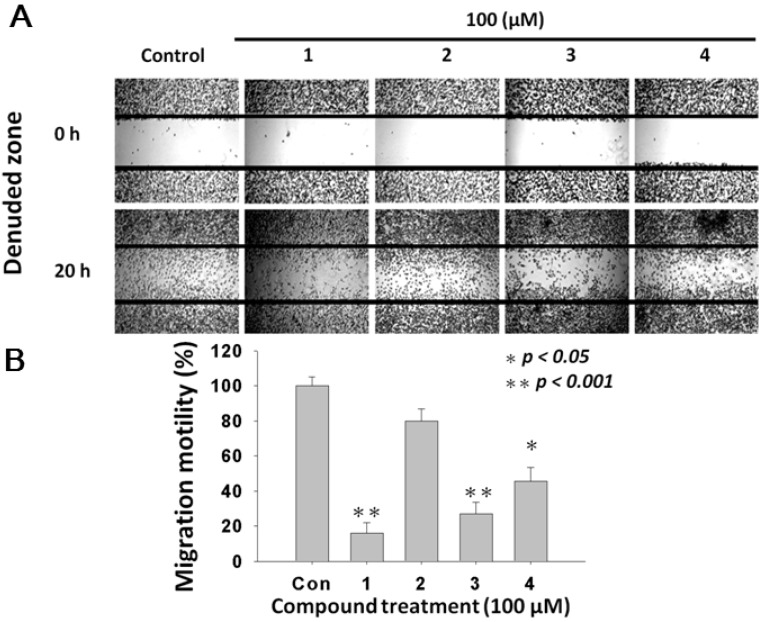
Effects of *L. tulipifera* compounds on migration of melanoma A375.S2 cells. (**A**) 5 × 10^5^ cells were seeded onto a 12-well plate and cells were scraped to create a clean 1-mm wide wound area within the confluent culture. Cells were treated with 100 μM of *L. tulipifera* compounds for 24 h. Afterwards, the wound gaps were photographed using an inverted phase-contrast microscopy. (**B**) The quantification analysis, * *p* < 0.01 for *L. tulipifera* compounds against vehicle, n = 3.

To investigate whether treatments of *L. tulipifera* compounds modulate the migration of melanoma cells, the wound-healing assay was performed. Figure 4A showed that the migration of A375.S2 melanoma cancer cells was inhibited by compounds **1**–**4**, as demonstrated by the denuded zones at 20-h post-treatment. Figure 4B demonstrated the quantitative analysis on the inhibition of migration ability by the four compound-treatments compared with the vehicle control. The calculated denuded zone (indicating the migratory ability of A375.S2 cells) for compounds were 100 ± 5%, 16.14 ± 6%, 79.8 ± 7%, 27.0 ± 6.6% and 45.5 ± 8%, respectively. Among these compounds, **2** and **3** exerted the most anti-migration effect. 

### 2.5. Compound ***3*** Causes a Dramatically Increased Level of Intracellular ROS

**Figure 5:**
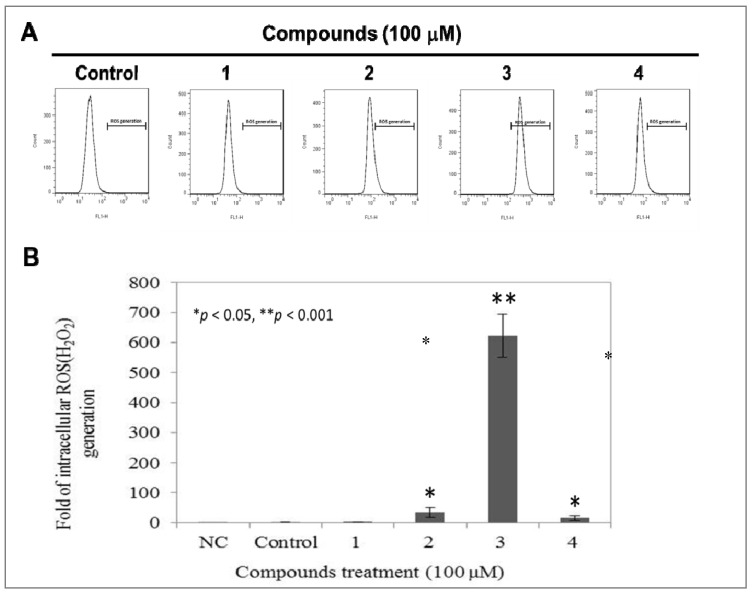
Modulations of endogenous ROS in A375.S2 cells by *L. tulipifera* compounds. 1 × 10^5^ of A375.S2 cells were seeded onto a 6-well plate and treated with or without 100 μM of **1**–**4** for 12 h, respectively. (**A**) The level of endogenous ROS was determined by DCFDA staining combined with a flow cytometry analysis. NC, Negative control, Unstained cells; Control, cells treated with vehicle. (**B**) Quantitative analysis. * *p* < 0.05 and ** *p *< 0.001 against vehicle control, respectively.

We further investigated whether *L. tulipifera* compounds modulate the level of intracellular ROS. Flow cytometer-based DCFDA staining, which is oxidized by ROS to the highly fluorescent DCF was used to detect the intracellular ROS, H_2_O_2_. The calculated effective ROS scavenge ability of compounds **1**–**4** were 1.0 ± 0.4%, 59.2 ± 81.9%, 34.594 ± 18.0%, 623.2 ± 71.8% and 15.6 ± 7.6%, respectively (Figure 5). Among these four compounds, compound **3** induced a dramatically increased level of ROS in A375.S2, whereas no significant change was observed in the other *L. tulipifera* compounds-treated cells. According to our above results, compound **3** exerts multiple bio-effects on A375.S2, including anti-migration, induction of G2/M accumulation and up-regulation of intracellular ROS without inhibiting the proliferation of A375.S2 cells, suggesting the chemoprevention potential of compound **3**.

Liriodenine derivatives have been reported to be cytotoxic to many cancer lines including breast cancer, lung cancer and hepatoma cells. Additionally, the anti-proliferative effects of liriodenine and its derivatives may mediate the apoptosis and ROS production within these cells [40,41,42,43]. Although *L. tulipifera* compounds **1**, **3** and **4** exert inhibitory effects on cellular proliferations and migrations of A375.S2 cells, only treatment with compound **3** causes a high level of intracellular ROS, whereas no significant changes of ROS were observed in compound **1** and **4**-treated cells. These observations indicate that the compound **1** and **4** inhibition of cellular proliferation and migration is ROS-independent. Previous studies indicated that the anti-cancer effects of liriodenine derivatives were achieved by inducing apoptosis [43]. In our study, at the dose of 100 μM, *L. tulipifera* compounds **1**, **3** and **4** displayed anti-proliferation (Figure 3) and anti-migration (Figure 4) potential but did not induce apoptosis. Interestingly, only compound **3** induced a definitely high level of intracellular ROS, however, there was still no detectable apoptotic cell deaths in compound **3**-treated cells (Figure 3). Accordingly, these above observations suggest that there might be novel anti-proliferation and anti-migration pathways triggered by *L. tulipifera.*

## 3. Experimental

### 3.1. General Procedures

UV spectra were obtained on a Jasco UV-240 spectrophotometer in MeCN. IR spectra were measured on a Hitachi 260-30 spectrophotometer (Hitachi, Tokyo, Japan). ^1^H-NMR (400/500 MHz) and ^13^C-NMR (100 MHz), HSQC, HMBC, COSY and NOESY spectra were obtained on a Varian (Unity Plus) NMR spectrometer (Varian, CA, USA). For each sample, 128 scans were recorded with the following settings: 0.187 Hz/point; spectra width, 14,400 Hz; pulse width, 4.0 μs; relaxation delay, 2 s. Low-resolution ESI-MS spectra were obtained on an API 3000 (Applied Biosystems, CA, USA) and high-resolution ESI-MS spectra on a Bruker Daltonics APEX II 30e spectrometer (Bruker, Bremen, Germany). Silica gel 60 (Merck, 70~230 mesh, 230~400 mesh) was used for column chromatography. Precoated silica gel plates (Merck, Kieselgel 60 F-254), 0.20 mm and 0.50 mm, were used for analytical TLC and preparative TLC, respectively, and visualized with 10% H_2_SO_4_.

### 3.2. Plant Material

The specimen of *L. tulipifera* was collected from Chiayi County, Taiwan in December, 2007. A voucher specimen was characterized by Dr. Jin-Cherng Huang of Department of Forest Product Compounds, Science and Furniture Engineering, National Chiayi University, Chiayi, Taiwan and deposited in the School of Medical and Health Sciences, Fooyin University, Kaohsiung County, Taiwan.

### 3.3. Extraction Isolation and Identification

The air-dried stems of *L. tulipifera* (9.0 kg) were extracted with MeOH at room temperature and the MeOH extract (187.5 g) was obtained upon concentration under reduced pressure. The MeOH extract was chromatographed over silica gel using CH_2_Cl_2_/MeOH as eluent to produce seven fractions. Among these seven fractions, part of fraction 4 was subjected to Si gel chromatography eluting with *n*-hexane/acetone to obtain (-)-glaucine (**3**) (19 mg) and (-)-norglaucine (**4**) (24 mg). Part of fraction 5 (7.43 g) was subjected to Si gel chromatography eluting with *n*-hexane/acetone to obtain liriodenine (**1**) (36 mg) and (-)-anonaine (**2**) (14 mg). These compounds were characterized by the comparison of their physical and spectral data (UV, IR, NMR and MS) with values given in the literature.

*Liriodenine* (**1**). Yellow needles (CH_2_Cl_2_); UV λ_max_: 224, 248, 265, 308 nm; IR ν_max_: 1625, 1022, 933 cm^−1^; ^1^H NMR (500 MHz, CDCl_3_): *δ* 6.38 (2H, *s*, -OCH_2_O-), 7.21 (1H, *s*, H-3), 7.59 (1H, *td*, *J* = 8.0, 1.5 Hz, H-9), 7.77 (1H, *td*, *J* = 8.0, 1.5 Hz, H-10), 7.79 (1H, *d*, *J* = 5.0 Hz, H-4), 8.60 (1H, *dd*, *J* = 8.0, 1.5 Hz, H-8), 8.67 (1H, *dd*, *J* = 8.0, 1.5 Hz, H-11), 8.90 (1H, *d*, *J* = 5.0 Hz, H-5); ESI-MS *m/z*: 275 [M]^+^.

*(-)-Anonaine *(**2**). Yellow powder (MeOH); UV λ_max_: 230, 272, 310 nm; IR ν_max_: 1040, 950 cm^−1^; ^1^H NMR (500 MHz, CDCl_3_): *δ* 2.66 (1H, *d*, *J* = 12.0 Hz, H-7α), 2.81 (1H, *t*, *J* = 14.0 Hz, H-7β), 2.93~3.06 (3H, *m*, H-4α, 4β, 5α), 3.40 (1H, *m*, H-5β), 3.98 (1H, *dd*, *J* = 14.0, 4.0 Hz, H-6α), 5.94 and 6.09 (each 1H, *d*, *J* = 1.0 Hz, -OCH_2_O-), 6.57 (1H, *s*, H-3), 7.21~7.33 (3H, *m*, H-8~10), 8.08 (1H, *d*, *J* = 8.0 Hz, H-11); ESI-MS *m/z*: 265 [M]^+^.

*(-)-Glaucine* (**3**). Brown powder (MeOH); UV λ_max_: 210, 282, 305 nm; IR ν_max_: 2800, 1600, 1580, 1310, 1105, 950 cm^−^^1^; ^1^H NMR (500 MHz, CDCl_3_): *δ* 2.63 (3H, *s*, N-CH_3_), 3.66 (3H, *s*, C_1_-OCH_3_), 3.89 (3H, *s*, C_2_-OCH_3_), 3.90 (3H, *s*, C_9_-OCH_3_), 3.93 (3H, *s*, C_10_-OCH_3_), 6.60 (1H, *s*, H-3), 6.78 (1H, *s*, H-8), 8.09 (1H, *s*, H-11); ESI-MS *m/z*: 355 [M]^+^.

*(-)-Norglaucine *(**4**). Brown powder (MeOH); UV λ_max_: 282, 303 nm; IR ν_max_: 2800, 1600, 1585, 1320, 1105, 975 cm^−^^1^; ^1^H NMR (500 MHz, CDCl_3_): *δ* 3.66 (3H, *s*, C_1_-OCH_3_), 3.90 (3H, *s*, C_2_-OCH_3_), 3.90 (3H, *s*, C_9_-OCH_3_), 3.93 (3H, *s*, C_10_-OCH_3_), 6.61 (1H, *s*, H-3), 6.78 (1H, *s*, H-8), 8.09 (1H, *s*, H-11); ESI-MS *m/z*: 341 [M]^+^.

### 3.4. Determination of DPPH·Radical Scavenging Capacity

DPPH· is a stable free radial with a violet color (absorbance at 517 nm) that changes its color to light yellow when the free radicals are scavenged [44]. Various concentrations of the four compounds were added to 0.1 mL of stable DPPH (60 μM) solution. When DPPH reacts with hydrogen-donating anti-oxidant, it is reduced, resulting in a decrease in absorbance at 517 nm. The analyzed time interval was 10 min per point, up to 30 min by using UV–vis spectrophotometer (BioTek Co.). Vitamin C was acted as a positive control. The DPPH· radical scavenging activity (%) was determined as:


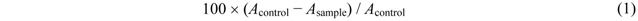


### 3.5. Metal Chelating Activity

The ferrous ion chelating potential of the four *L. tulipifera *compounds was investigated according to a previously described method [44]. Brieﬂy, various test concentrations of samples dissolved in DMSO were added to a solution of 2 mM FeCl_2_·4H_2_O (0.01 mL). The reaction was initiated by the addition of 5 mM ferrozine (0.02 mL), and the mixture was vigorously shaken and left standing at room temperature for 10 min. The absorbance of the mixture was then read at 562 nm against a blank. EDTA was used as a positive control.

### 3.6. Reducing Power

The reducing powers of our natural pure compounds were determined according to the method of [44]. Briefly, various concentrations of test samples were mixed with 67 mM phosphate buffer (pH 6.8, 0.085 mL) and 20% potassium ferricyanide [K_3_Fe(CN)_6_, 2.5 μL) The mixture was incubated at 50 °C for 20 min, and trichloroacetic acid (10%, 0.16 mL) was then added to the mixture that was then centrifuged for 10 min at 3,000 g. The upper layer of the solution (75 μL) was mixed with 2% FeCl_3_ (25 μL), and the absorbance was measured with a 96-well plate spectrophotometer at 700 nm. BHA was used as a positive control. A higher absorbance demonstrates a higher reductive capability.

### 3.7. Assay on Mushroom Tyrosinase Activity

Tyrosinase inhibitory activity was determined spectrophotometrically according to the method described previously [45], with minor modifications. Assays were conducted in a 96-well microplate, an ELSA plate reader (Molecular Devices) being used to determine the absorbance at 490 nm. Kojic acid was used as a positive control. The test substance was dissolved in aqueous DMSO, and incubated with L-tyrosine (2.5 mg/mL) in 50 mM phosphate buffer (pH 6.8). Then, 25 U/mL of mushroom tyrosinase in the same buffer was added, and the mixture was incubated at 37 °C for 30 min. Tyrosinase inhibitory activity was determined at 490 nm by the following equation:


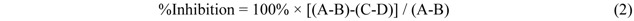


where A is the optical density (OD_490_) without test substance; B is the OD_490_ without test substance, but with tyrosinase; C is the OD_490_ with test substance; and D is the OD_490_ with test substance, but without tyrosinase. The results are listed in Table 1. 

### 3.8. Cell Culture

Human melanoma cell lines A375.S2 were obtained from the American Type Cell Culture Collection (ATCC, Manassas, VA, USA). It was maintained in monolayer culture at 37 °C and 5% CO_2_ in DMEM supplemented with 10% FBS, 10 μg/mL of penicillin, 10 μg/mL of streptomycin, and 0.25 μg/mL of amphotericin B.

### 3.9. Cell Viability Assay–MTT Assay

The MTT assay was used to determine cell viability and proliferation. The cell lines were seeded in 96-well culture plates (1 × 10^4^ cells/well). After seeding cells for 24 h, various compounds with concentration 100 μM were added. Within 24 h of compound treatments, images of human melanoma A375.S2 cells were taken at suitable time intervals. MTT solution (5 mg/mL and dissolved in phosphate buffered saline; PBS) was diluted 1:10 in culture medium and added to a culture dish followed by an incubation at 37 °C. After 2 h of MTT treatment, the media was removed and each precipitate in a specific dish was dissolved in 100 μL of DMSO to dissolve the purple formazan crystals. After the dishes were gently shaken for 20 min in the dark to ensure maximal dissolution of formazan crystals, the optical density (OD) values of the supernatant were measured at 595 nm. All experiments were repeated at least three times. In consideration of the possible anti-proliferative effects of DMSO, a maximal amount (0.5%) of DMSO was added to culture and used as positive controls. DMSO at this amount was found not to affect the growth of the human melanoma A375.S2 cells.

### 3.10. Assessment of Cell Cycle Distribution

The cell cycle distribution was determined by propidium iodide (PI) staining as described previously [46]. Briefly, 1 × 10^6^ cells were treated with DMSO as vehicle control or 100 μM of *L. tulipifera* compounds for 24 h. After treatments, cells were harvested, washed twice with PBS and fixed in 70% ethanol. After centrifugation at 3000 rpm for 5 min at 4 °C, the cell pellets were stained with 10 μg/mL PI (Sigma, St. Louis, MO, USA) and 10 μg/mL RNase A in PBS for 30 min at 37 °C in the dark. The cells were analyzed using a FACScan flow cytometer (Becton-Dickinson, Mansfield, MA, USA) and the results were analyzed using the Cell-Quest software (Becton-Dickinson).

### 3.11. Wound Healing Assay

The wound healing assay was described previously [46]. In brief, a total of 3 × 10^5^ A375.S2 cells were seeded onto 12-well plates, treated with PBS (as vehicle control) or indicated concentrations of *L. tulipifera* compounds, and then grown to complete confluence. A 200-μL plastic pipette tip was used to scratch the culture monolayer and create a clean 1-mm-wide wound area in the A375.S2 confluent culture. After further incubation at 37 °C for 16 h, the wound gaps were photographed. The wound areas were then analyzed and calculated using the software “TScratch” (http://www.cse-lab.ethz.ch). The migration motility of cells was determined as % of vehicle control cells.

### 3.12. Determination of Intracellular ROS

It has been reported that the intracellular level of ROS is associated with the inhibition of proliferation and cellular migration by a variety of stresses, including anti-cancer agents. Therefore, to determine whether the anti-proliferative effect of *L. tulipifera* compounds involves the production of oxidative stress, the changes in endogenous ROS levels were detected using a fluorescent dye DCFDA (Sigma-Aldrich). A total of 1 × 10^5^ A375.S2 cells were seeded onto a 6-well culture plate, treated with or without *L. tulipifera* compounds for 12 h, respectively. Afterwards, cells were harvested and stained with 100 nM DCFDA for 30 min at 37 °C in PBS, then washed twice with PBS. The fluorescence of DCFDA was measured by a flow cytometer. The excitation wavelength of DCFDA is 485 nm, and the emission wavelength is 530 nm.

### 3.13. Statistical Analysis

All data are the means ± SD from at least triplicate experiments. The signiﬁcance of the differences was analyzed by a one-way analysis of variance (ANOVA), with *p* < 0.05 or 0.01 as considered significant.

## 4. Conclusions

The study reports the antioxidant, mushroom tyrosinase inhibition and anticancer properties of four pure constituents from *L. tulipifera. *Our results demonstrated that *L. tulipifera* compounds may play an assistant role in the anti-oxidant and anti-tyrosinase effects. We also demonstrated the multiple anti-cancer effects of compounds **1**–**4**. Importantly, among these four compounds, compound **3** displayed a significant anti-migration potential with minor cytotoxicity against human melanoma A375.S2 cells. These data suggested that *L. tulipifera *components may be promising chemopreventive agents against melanoma metastasis in clinical applications. The mechanism underlying the anti-cancer effect of *L. tulipifera *compounds will be examined in our further study. To our best knowledge, this is the first study to demonstrate the anti-migration effect of liriodenine derivatives isolated from *L. tulipifera* without inducing apoptosis.
